# Artificial Intelligence for Predictive Microbiome Engineering: Integrating Community Design and Environmental Validation

**DOI:** 10.1111/1758-2229.70454

**Published:** 2026-10-29

**Authors:** Mohammad Nazrul Islam Bhuiyan, Sheikh Julfikar Hossain

**Affiliations:** ^1^ BCSIR Chattogram Laboratories, BCSIR Chattogram Bangladesh; ^2^ Institute of Food Science and Technology, Bangladesh Council of Scientific and Industrial Research (BCSIR) Dhaka Bangladesh; ^3^ Biotechnology and Genetic Engineering Discipline, Life Science School Khulna University Khulna Bangladesh

**Keywords:** artificial intelligence, mechanistic modelling, microbial ecology, microbiome engineering, synthetic microbial communities

## Abstract

Microbial ecosystems can now be measured across genomic, transcriptomic, proteomic, metabolomic, and environmental dimensions, yet their emergent functions remain difficult to predict and control. Artificial intelligence (AI) can integrate heterogeneous data, but predictive accuracy alone does not establish causality, transferability, or controllability. This review developed a five‐layer framework: observe, infer, predict, design, and learn that connects microbial measurement to prospective intervention and experimental feedback. We critically synthesise evidence from sequence and protein learning, multi‐omics integration, metabolic modelling, synthetic microbial communities, environmental biotechnology, and temporal forecasting. Recent primary studies illustrate progress in forecasting community trajectories, predicting microbial competition and designing consortia for pollutant degradation; they also reveal important boundaries between model performance and demonstrated ecosystem control. We examine risks arising from compositional data, information leakage, dependent observations, batch effects, uncertain calibration, sparse perturbation data, ecological instability, and evolutionary change. We propose operational criteria for ecosystem programmability and a staged validation pathway from controlled systems to field‐relevant environments. Progress will depend on standardised context‐rich data, hybrid AI–mechanistic models, prospective benchmarks, independent replication, and safety‐constrained design.

## Graphical abstract

Overview of an artificial intelligence (AI)‐enabled framework for predictive microbiome engineering, integrating multi‐omics and environmental data through five iterative stages: observe, infer, predict, design, and learn. Data‐driven predictions are linked to experimental validation and potential environmental applications, with data quality, mechanistic plausibility, uncertainty, ecological stability, and biosafety guiding responsible deployment.

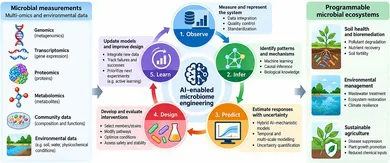

## 1 Introduction

Microbial ecosystems underpin biogeochemical cycling, pollutant transformation, soil fertility, and host‐associated functions, yet predicting their responses to environmental change or deliberate intervention remains challenging. Advances in high‐throughput sequencing, metagenomics, transcriptomics, proteomics, and metabolomics have expanded microbial characterisation, while machine‐learning (ML) supports microbial classification, functional annotation, biomarker discovery, and environmental monitoring (Eraslan et al. 2019; Marcos‐Zambrano et al. 2021). However, retrospective classification and predictive accuracy alone do not establish causal mechanisms or demonstrate that an intervention can reproducibly achieve a desired ecosystem function. These limitations are especially important in environmental microbiology, where resource availability, physicochemical conditions, spatial heterogeneity, and interactions with indigenous communities shape microbial activity (Lopatkin and Collins 2020).

Existing research and reviews address complementary parts of this challenge. Microbiome‐focused ML research emphasises classification, feature selection, and phenotype prediction, while protein‐learning approaches improve molecular‐function annotation (Bepler and Berger 2021; Bileschi et al. 2022). Genome‐scale metabolic models connect genomic and omics data with biochemical constraints, and neural–mechanistic approaches seek to improve metabolic predictions (Faure et al. 2023). Synthetic‐biology studies demonstrate the engineering of selected microbial traits and communities (Charbonneau et al. 2020). Nevertheless, these approaches often evaluate different endpoints, ranging from molecular annotation and metabolic flux to community dynamics and process performance. Consequently, success at one level cannot be assumed to demonstrate predictable community behaviour, controllability, or ecological stability. The specific gap is the lack of a unified, evidence‐oriented structure that connects these capabilities to prospective intervention, experimental validation, and environmental deployment.

Recent primary studies illustrate both progress and remaining limitations. MicroProphet forecasts microbial abundance trajectories from incomplete longitudinal data, including selected synthetic and host‐associated communities (Zhang et al. 2025). However, forecasting observed trajectories does not necessarily predict responses to deliberate perturbation. MIND uses translation‐related functional measurements to infer substrate preferences and microbial competition, supporting targeted microbiome modulation in the tested systems (Moyne et al. 2026). In environmental biotechnology, GENIA combines cultivation, pollutant‐specific screening, genomic information, and ML to guide synthetic‐community design for pollutant degradation (De la Vega‐Camarillo et al. 2026). Its laboratory and soil‐microcosm results provide evidence of computationally guided community design, but do not establish field‐scale efficacy, long‐term persistence, or the absence of unintended ecological effects. Collectively, these studies demonstrate distinct advances in temporal forecasting, interaction‐informed intervention, and environmental consortium design, not universal programmability of complex microbial ecosystems.

Translating these advances into reliable applications also requires rigorous attention to data leakage, non‐independent observations, compositionality, batch effects, meaningful baselines, and uncertainty calibration. Environmental validation must measure relevant functions directly and assess performance under realistic variability. Depending on the application, endpoints may include pollutant transformation, functional stability, persistence, non‐target effects, and reproducibility across environmental settings.

To address these gaps, this review proposes an observe–infer–predict–design–learn framework that connects microbial measurement and data representation with mechanistic inference, prospective prediction, intervention design, and experimental feedback. We define a programmable microbial ecosystem as a community for which a target function can be specified, prospectively predicted, induced through a defined intervention, reproduced under stated conditions, and iteratively improved using experimental feedback. This is a proposed operational definition, not a claim that complex natural microbiomes are already fully programmable. The framework's novelty lies in linking these five stages to explicit evidence requirements and distinguishing designability, constructing a candidate community that performs a desired function under defined conditions from deployability, maintaining that function safely and reproducibly in the intended environment. By integrating primary‐study evidence, methodological safeguards, and staged environmental validation, this review provides a basis for evaluating when AI‐enabled microbiome research moves beyond descriptive analysis towards experimentally supported and ecologically responsible engineering.

## 2 Review Approach and Scope

This article is a critical narrative review rather than a systematic review or meta‐analysis. The literature search focused on publisher platforms, PubMed, Google Scholar, Scopus, and open‐access repositories to identify recent primary studies directly relevant to microbial community‐level prediction, perturbation, and environmental deployment. Priority was given to primary research reporting clearly defined models, explicit validation strategies, and biological or ecological outcomes, as well as methodological studies addressing data structure and model evaluation. Broader applications of artificial intelligence were included only when they provided relevant transferable methods or informed safety considerations for microbial prediction and engineering.

Evidence was interpreted using four questions: (1) What are predicted molecular properties, community composition, trajectory, or ecosystem function? (2) Is evaluation retrospective, held‐out, external, or prospective? (3) Does the study perturb the system and test whether the intervention changes the predicted outcome? (4) What are the boundaries of transferability, stability, and safety? Because studies use different organisms, endpoints, sampling schedules, and metrics, their performance values are not pooled or ranked as if directly comparable. Table 1 is therefore an evidence map, not a quantitative meta‐analysis. This approach preserves distinctions among prediction, mechanistic explanation, and intervention efficacy.

**TABLE 1:** Comparative evidence from studies relevant to predictive microbiome engineering.

Study/evidence type	Biological system	Prediction or design target	Validation design and reported outcome	Main limitation/evidence boundary
MicroProphet; temporal forecasting (Zhang et al. 2025)	Synthetic microbial communities; longitudinal human gut, infant and postmortem datasets	Forecast microbial abundance trajectories from incomplete time series	Evaluated against benchmark models; community/subject‐level splits were used in relevant analyses. Reported accurate forecasts using the initial ~30% of time points in several datasets.	Forecasting trajectories under observed conditions does not establish causal control of trajectories under new interventions; performance and endpoints vary across datasets.
MIND; functional prediction plus intervention (Moyne et al. 2026)	Defined microbial communities and host‐associated microbiomes; in vitro and in vivo tests	Predict competition and substrate preferences from functional measurements; guide targeted modulation	The study linked translation‐related functional measurements and community profiles to predictions of resource use and competition, then tested targeted prebiotic/probiotic‐style interventions.	Transfer depends on nutrient context, strain composition and host/environment; not evidence that arbitrary complex ecosystems are fully programmable.
GENIA; AI‐guided synthetic‐community design (De la Vega‐Camarillo et al. 2026)	Environmental isolates; pollutant degradation in minimal medium and soil microcosms	Select a consortium for multi‐pollutant degradation using genome‐informed features and graph‐based models	A nine‐member consortium was tested for lignin, atrazine and PFOS degradation; the authors reported > 70% degradation at 3 weeks in soil microcosms.	Microcosm evidence is stronger than flask‐only evidence but does not establish performance across field soils, climates, long‐term persistence or non‐target effects.
Metagenomic ML; retrospective prediction (Pasolli et al. 2016)	Large, heterogeneous metagenomic datasets	Classify phenotypes and identify microbial signatures	Cross‐dataset machine‐learning analyses demonstrated that large metagenomic collections can support prediction and biological insight.	Retrospective classification does not establish intervention response; dataset composition and study‐specific signals can affect generalisation.
Antibiotic‐resistance gene prediction (Arango‐Argoty et al. 2018)	Metagenomic sequences	Identify likely antimicrobial‐resistance genes	DeepARG provided a deep‐learning workflow for resistance‐gene prediction from metagenomic data.	Predicted gene presence is not equivalent to gene expression, resistance phenotype, transmission risk or ecosystem‐level treatment effect.
Hybrid metabolic prediction (Faure et al. 2023; Gonçalves et al. 2023)	Genome‐scale metabolic models and omics‐informed systems	Estimate metabolic fluxes or pathway activity	Hybrid neural–mechanistic modelling and omics‐based flux prediction support integration of learned patterns with biochemical constraints.	Model outputs depend on pathway completeness, data quality and assumptions; community‐scale intervention effects require separate validation.
AI‐guided synthetic‐biology design (Radivojević et al. 2020; Sabzevari et al. 2022)	Synthetic‐biology design spaces and engineered strains	Prioritise designs and optimise strain‐level objectives	Automated recommendations and reinforcement‐learning approaches show how model‐guided search can narrow candidate designs.	Design‐space optimisation does not automatically transfer to multispecies ecology or environmental deployment.

## 3 From Measurements to Computational Representations

### 3.1 Molecular Measurements and Biological Context

The observation layer establishes the biological and environmental information available for inference and prediction. It may include genome sequences, transcripts, proteins, metabolites, microbial phenotypes, community composition, and environmental measurements such as temperature, pH, moisture, nutrient availability, and pollutant concentration. These data represent different biological scales and timescales: genome sequences generally describe relatively stable genetic potential, whereas transcription, protein abundance, metabolite concentrations, and microbial activity can change substantially with environmental conditions. Their integration can narrow the gap between encoded potential and realised function, but only when the measurements are linked to the same biological samples, relevant time points, compartments, and experimental conditions. Multi‐omics integration should therefore be treated as a problem of biological alignment and interpretation, not simply the concatenation of feature matrices (Bennett et al. 2023; Zhang et al. 2024; Yetgin 2025).

Protein language models and deep‐learning methods extend the range of sequence representations available for microbial research. By learning statistical patterns in protein sequences, these methods can support functional annotation, identify candidate proteins, and prioritise sequences for experimental investigation (Bepler and Berger 2021; Bileschi et al. 2022; Chandra et al. 2023). Their value is particularly clear where sequence databases are incomplete or conventional similarity‐based annotation provides limited information. However, a predicted annotation remains an inference rather than a direct measurement of biochemical activity. Performance may also be weaker for proteins or taxa that are poorly represented in training data. In environmental microbiology, this limitation matters because uncommon or poorly characterised organisms may contribute disproportionately to functions under specific conditions. Functional predictions should therefore carry information about their confidence and applicability domain, and should be validated experimentally when they determine subsequent community‐design decisions.

Genome‐resolved metagenomics provides a further connection between molecular measurements and community context. Reconstructed metagenome‐assembled genomes can associate candidate organisms with genes, pathways, and potential biogeochemical functions. METABOLIC, for example, provides a computational framework for inferring metabolic and biogeochemical traits from microbial genomes and community genomic data (Zhou et al. 2022). Such approaches help identify potential contributors to processes such as nutrient cycling and environmental transformation, but their outputs depend on genome recovery, completeness, contamination, annotation quality, and reference‐database coverage. Incomplete or missing annotations do not necessarily indicate the absence of a biological function. A model that interprets unannotated pathways as biologically absent may systematically underestimate the capabilities of poorly characterised environmental taxa.

For predictive engineering, the resulting representation should distinguish at least three types of information: directly measured variables, computationally inferred properties, and experimentally supported functions. This separation helps prevent uncertain molecular annotations from being treated as established biological facts in downstream models. It also makes uncertainty traceable: researchers can identify whether a failed prediction arose from inadequate measurements, incorrect functional annotation, an incomplete mechanistic representation, or an inaccurate model of community behaviour.

### 3.2 Compositionality, Absolute Abundance and Technical Variation

Sequencing‐based microbial profiles are usually compositional because the observed counts are constrained by the sequencing process and are often converted into relative‐abundances. Consequently, the proportion assigned to one taxon can increase simply because other taxa decline, even when the first taxon's absolute abundance remains unchanged. Conventional correlations and regression analyses applied without accounting for this constraint can produce misleading associations and distorted network structures (Gloor et al. 2017). This is not a minor preprocessing issue: it can affect the inferred interactions, candidate biomarkers, and ecological mechanisms subsequently used to design interventions.

Analyses should therefore use methods appropriate to compositional data, document how zero values and low‐prevalence taxa are handled, and state the assumptions underlying normalisation and transformation. Where feasible, relative profiles should be complemented by quantitative measurements such as spike‐in standards, flow cytometry, quantitative PCR, or biomass estimates. These measurements are not interchangeable, and each has its own limitations, but they can help distinguish changes in relative composition from changes in absolute microbial abundance. When absolute measurements are unavailable, the manuscript should acknowledge that limitation rather than interpreting relative abundance changes as direct evidence of population growth or decline.

Technical variation creates a second major challenge. Extraction protocols, primer selection, sample storage, sequencing platform, library preparation, sequencing depth, and bioinformatic processing can all affect the observed microbial profile. Models may consequently learn laboratory, batch or study‐specific signatures instead of biologically meaningful patterns, particularly when treatment groups, environmental sites or outcome classes are confounded with processing batches. This risk is substantial when datasets are combined across studies or environmental settings.

A robust observation pipeline should record sample handling, extraction and library protocols, sequencing depth, technical replicates, relevant environmental covariates and other metadata needed to interpret variation. Preprocessing, feature selection, normalisation and hyperparameter tuning should be performed within the training procedure rather than using information from held‐out observations. Otherwise, data leakage can inflate apparent predictive performance. Validation should also respect the intended prediction task: if the objective is to predict outcomes in a new site, community, or study, splitting individual samples randomly may produce an overly optimistic estimate because related observations can occur in both training and test sets (Pasolli et al. 2016; D'Urso and Broccolo 2024). Microbiome‐specific best‐practice guidance, including the framework described by Papoutsoglou et al. (2023), further supports transparent reporting of data‐ processing and reproducible analysis workflows.

These safeguards should be considered part of model development rather than optional checks performed after training. A model cannot provide reliable evidence of ecosystem predictability if its apparent performance depends on technical signatures, non‐independent observations, or leakage from the test data.

### 3.3 Multimodal Integration Without False Certainty

Integrating genomics, transcriptomics, proteomics, metabolomics, and environmental measurements can reveal relationships that are difficult to identify from any single data layer. Nevertheless, cross‐layer association does not automatically establish a causal pathway. A metabolite may correlate with a taxon because both respond to substrate availability; a transcript may indicate stress without being the cause of a community‐level phenotype; and a predicted metabolic pathway may not be active under the sampled conditions. Integration increases the amount of information available to a model, but it can also increase redundancy, propagate measurement error, and amplify biases shared across datasets.

For this reason, multimodal models should specify which variables are directly measured, which are inferred, and which relationships have been tested experimentally. Predictive utility should be evaluated separately from mechanistic interpretation. A model may forecast an outcome accurately without identifying the correct causal mechanism, while a biologically plausible mechanism may fail to provide useful prospective predictions. Where feasible, candidate relationships should be evaluated using targeted perturbations, time‐resolved measurements, appropriate negative controls, and independent replication.

The degree of contextual information required also increases with the complexity of the target. A protein‐function prediction may be evaluated against biochemical evidence; a metabolic prediction requires relevant substrates and physiological conditions; and a community‐level prediction must account for interactions, environmental constraints, and the functional endpoint of interest. Figure 1 should illustrate this progression from molecular measurements to organismal activity, community interactions, and ecosystem function, emphasising that each transition introduces additional assumptions and validation requirements.

**FIGURE 1:**
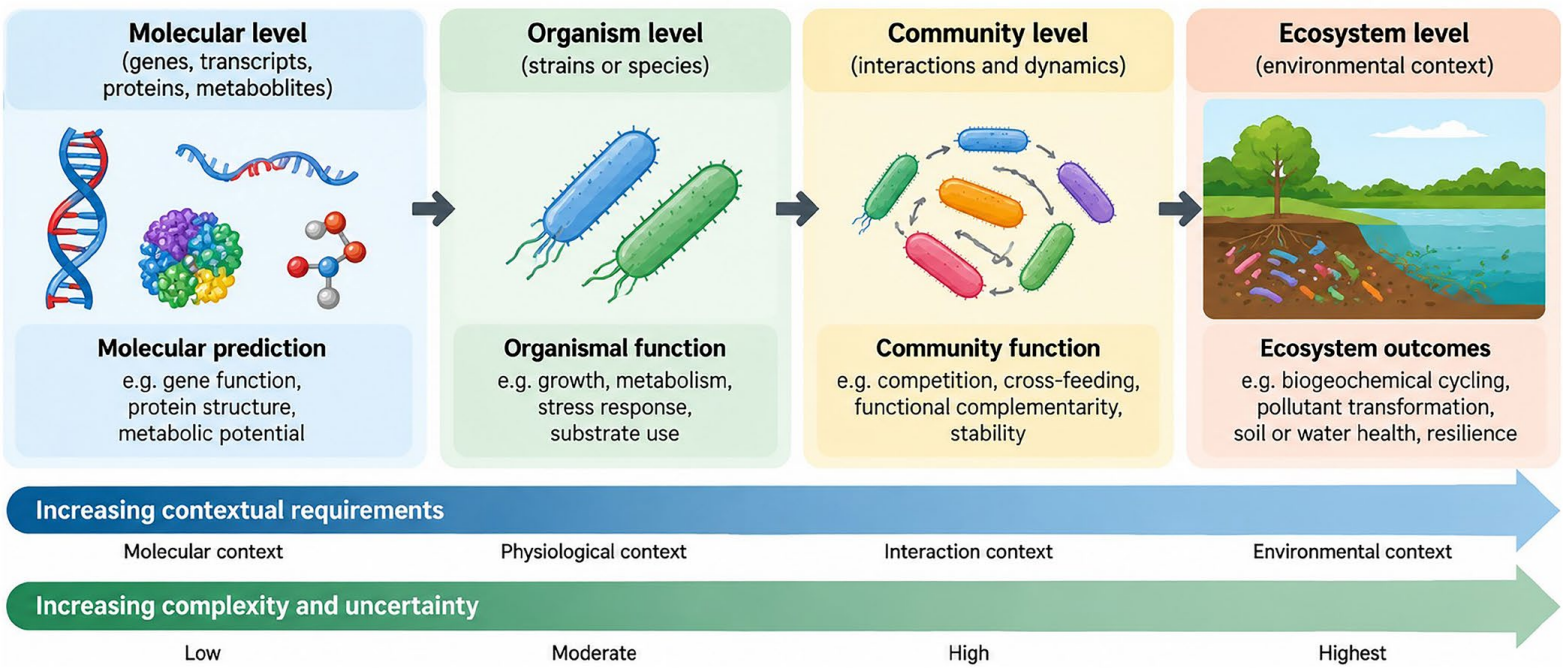
From microbial measurements to ecosystem‐level prediction. Conceptual hierarchy linking molecular measurements to organismal function, microbial community interactions, ecosystem outcomes, and responses to intervention. Biological context, interacting components, and uncertainty generally increase across these levels. Molecular‐level predictive accuracy alone does not establish community function, ecological persistence, or intervention effectiveness.

## 4 Inferring Mechanisms and Predicting Community Dynamics

### 4.1 Association, Mechanism and Causal Evidence

The inference layer converts observed data into candidate biomarkers, ecological interactions, and mechanistic explanations. Statistical learning, feature‐importance measures, attention mechanisms, SHAP values, and inferred interaction networks can help prioritise hypotheses for further testing. However, these tools do not independently establish causality. A feature may be predictive because it is correlated with an unmeasured driver, and an inferred interaction may reflect shared responses to environmental conditions rather than a direct biological relationship. Compositional constraints, sampling bias, and indirect effects can further complicate the interpretation of microbial association networks (Gloor et al. 2017; Papoutsoglou et al. 2023).

The distinction is important because predictive microbiome engineering seeks not only to identify organisms associated with a function, but also to determine whether changing a selected organism, resource, or environmental condition will alter that function. Feature importance identifies variables useful to a model under its evaluation conditions; it does not necessarily identify variables whose manipulation will change the outcome. Similarly, a plausible metabolic pathway does not establish that the pathway is active or rate‐limiting in the community being studied.

Stronger mechanistic evidence requires experiments that connect a specified intervention to a measurable response. Investigators should define the intervention, comparator, primary endpoint, observation period, and relevant alternative explanations before testing a hypothesis. Depending on the system, appropriate experiments may include strain addition or removal, substrate manipulation, targeted gene perturbation, environmental modification, or controlled community reconstruction. Time‐resolved measurements can help establish whether the predicted intermediate changes precede the functional outcome, while negative controls and independent replication help identify alternative explanations.

Where experimental manipulation is impractical, the analysis should remain explicit about the limits of causal inference. Models may still provide useful predictions without fully identifying the underlying mechanism, but such predictions should not be described as validated causal relationships. For environmental applications, this distinction matters because a model that identifies a taxon associated with pollutant removal does not necessarily establish that adding the taxon will improve remediation under field conditions.

### 4.2 Hybrid AI–Mechanistic Models

Data‐driven models can represent nonlinear relationships and interactions among large numbers of features, but they may extrapolate poorly beyond the conditions represented in their training data. Mechanistic models encode biochemical or ecological constraints, yet they can be limited by incomplete pathway knowledge, uncertain parameters, computational demands, and the difficulty of representing complex communities. Hybrid approaches seek to combine the pattern‐recognition capacity of ML with the explanatory structure of mechanistic models.

Faure et al. (2023) demonstrated a neural–mechanistic approach that improved the predictive power of genome‐scale metabolic models, illustrating how learned components can be integrated with established metabolic structure. Related work on ML‐based metabolic prediction also illustrates opportunities to connect omics measurements with biochemical estimates (Gonçalves et al. 2023). These studies support the use of hybrid approaches for selected prediction tasks; they do not establish that every hybrid model will outperform a well‐calibrated statistical or mechanistic baseline.

At community‐scale, hybrid models could combine genome‐scale metabolic networks, resource competition, growth kinetics, environmental covariates, and measured community dynamics. ML components might estimate uncertain parameters, represent poorly characterised residual relationships, or identify candidate interactions, while mechanistic components impose known mass‐balance or biochemical constraints. Such models could be useful for predicting community responses to nutrient changes, pollutant exposure, or the introduction of selected strains.

However, including mechanistic components does not guarantee biological validity. Incomplete reaction networks, unmeasured resources, incorrect kinetic assumptions, and poorly represented environmental conditions can still produce misleading predictions. A credible hybrid model should therefore make its assumptions explicit, assess sensitivity to uncertain parameters, compare its performance with simpler alternatives, and test predictions under conditions excluded from calibration. When a model is intended to guide intervention, prospective experimental validation is more informative than improved fit to the data used to construct it.

### 4.3 Temporal Forecasting and Incomplete Observations

Microbial communities change over time in response to environmental fluctuations, resource availability, biological interactions, and disturbance. Cross‐sectional data can identify associations, but they provide limited evidence about succession, response rates, resilience, or stability. Longitudinal data are therefore important for understanding community dynamics and developing predictive models. They also introduce additional statistical challenges, including irregular sampling intervals, missing observations, subject‐ or site‐specific trajectories, and dependence among repeated measurements.

Validation design must reflect these dependencies. Randomly allocating individual time points from the same community or subject to training and test sets can allow information about a trajectory to appear in both sets, producing overly optimistic estimates of predictive performance. If the intended application is forecasting for a new community, subject, site, reactor, or study, validation should separate those independent units accordingly. If the intended application is forecasting future observations from a known system, the evaluation should instead preserve the temporal ordering and ensure that future observations do not influence model fitting or preprocessing. The appropriate split therefore depends on the intended use of the model, rather than on a universal rule for all longitudinal datasets (Papoutsoglou et al. 2023).

MicroProphet provides a recent example of temporal forecasting from incomplete microbiome observations. Zhang et al. (2025) developed a temporal‐aware Transformer framework that reconstructs microbial abundance trajectories using an initial portion of the observed time series without requiring explicit data imputation. The authors evaluated the model across synthetic communities, human gut microbiome datasets, infant gut development, and postmortem microbial succession. Their results indicate that useful trajectory forecasts can be obtained from incomplete longitudinal observations, including evaluations in which the initial 30% of time points were used as input. These findings support temporal modelling as a promising component of predictive microbiome research, although performance remains tied to the datasets, prediction targets, and evaluation procedures studied.

The key limitation is the distinction between forecasting under an observed regime and predicting the response to a new intervention. A model may forecast future abundance trajectories accurately when the system continues under conditions similar to those observed during training, yet fail when a nutrient is introduced, a strain is added, an antibiotic is applied, or an environmental disturbance changes the governing dynamics. Such interventions may alter competition, resource availability, interaction strengths, or the relationship between community composition and function. A forecast alone therefore does not demonstrate intervention controllability.

To move from forecasting towards predictive engineering, temporal models should be evaluated in prospective perturbation experiments. The predicted response should be recorded before the intervention, compared with an appropriate control, and assessed using both community‐level measurements and the functional endpoint of interest. Prediction intervals, calibration, and failure cases should be reported alongside point estimates. Where possible, independent communities or environmental conditions should be used to test transferability. These requirements would help establish whether temporal forecasting can become a reliable component of an iterative engineering process rather than remain primarily a tool for extrapolating observed dynamics.

## 5 Designing Interventions From Predictions

### 5.1 From Candidate Ranking to Intervention

The design layer translates model predictions into testable interventions, including community‐member selection, nutrient modification, strain engineering, and process optimisation. Objectives may include pollutant degradation, nutrient recovery, pathogen suppression, or improved process stability. Candidate selection should balance target performance with reproducibility, resource requirements, ecological stability, and safety rather than optimise a single outcome under laboratory conditions.

Machine‐learning‐assisted tools can prioritise experimental designs, as demonstrated by Radivojević et al. (2020). However, computational ranking does not establish biological efficacy. Reliable evaluation requires predefined targets, appropriate comparators, independent biological replicates, and prospective testing. Failed predictions should also be reported because they can reveal missing mechanisms or environmental dependencies.

Importantly, a candidate optimised in a defined medium may perform differently in soil or wastewater because of substrate availability, indigenous competitors, and physicochemical variation. Design objectives and validation conditions should therefore reflect the intended application.

### 5.2 Predicting Competition and Substrate Preference

MIND, developed by Moyne et al. (2026), uses measurements of mRNA translation prioritisation to infer microbial substrate preferences and competitive interactions. Its applications to synthetic communities, soil, human faecal samples, and a mouse model illustrate how functional measurements can inform targeted microbiome modulation. Unlike taxonomic association alone, this approach links measured microbial activity with hypotheses about ecological interactions.

Nevertheless, MIND does not establish that all microbial interactions are predictable from translation‐related measurements. Facilitation, antagonism, phage predation, spatial structure, and environmental stress may introduce mechanisms beyond the model's scope. Predictions should therefore be tested under defined conditions and independently evaluated before transfer to different soils, hosts, or reactors is assumed.

### 5.3 Synthetic Microbial Communities and Design Trade‐Offs

Synthetic microbial communities (SynComs) provide a tractable means of investigating cross‐feeding, competition, facilitation and division of metabolic labour. Their applications include agriculture, remediation and industrial biotechnology, but performance depends on community composition and environmental conditions (Contreras‐Salgado et al. 2024; Mehlferber et al. 2024).

Community design requires balancing functional complementarity, in which members perform different but coordinated functions, against redundancy, which may help preserve function when individual members decline. Increasing community complexity can also increase interaction uncertainty and reduce reproducibility. A larger consortium is therefore not necessarily a better one.

A practical strategy is to begin with a small, mechanistically justified community, measure functional output and abundance under relevant conditions, and test stability under specified disturbances. Validation should then progress to more representative environmental matrices. Recent guidance on rigorous SynCom design likewise emphasises systematic construction, ecological assessment, and application‐specific validation (Zhang et al. 2026).

### 5.4 Closed‐Loop Learning and Automated Experimentation

The learning layer compares experimental outcomes with predictions to update models, refine candidate designs, and prioritise subsequent experiments. Active learning can select experiments that reduce uncertainty or distinguish competing hypotheses. Automated cultivation and high‐throughput screening may improve experimental throughput, but reliable learning still requires standardised procedures, traceable metadata, and reproducible measurements.

Figure 2 summarises the design–build–test–learn (DBTL) cycle: researchers define a target and constraints, rank candidates computationally, test selected interventions against appropriate controls, compare observations with predictions, and update the model. Recording uncertainty and unsuccessful candidates is essential to prevent selective reporting and improve subsequent decisions. This cycle is a proposed workflow; it should not be interpreted as evidence that all current microbiome‐engineering studies implement a complete closed‐loop.

**FIGURE 2:**
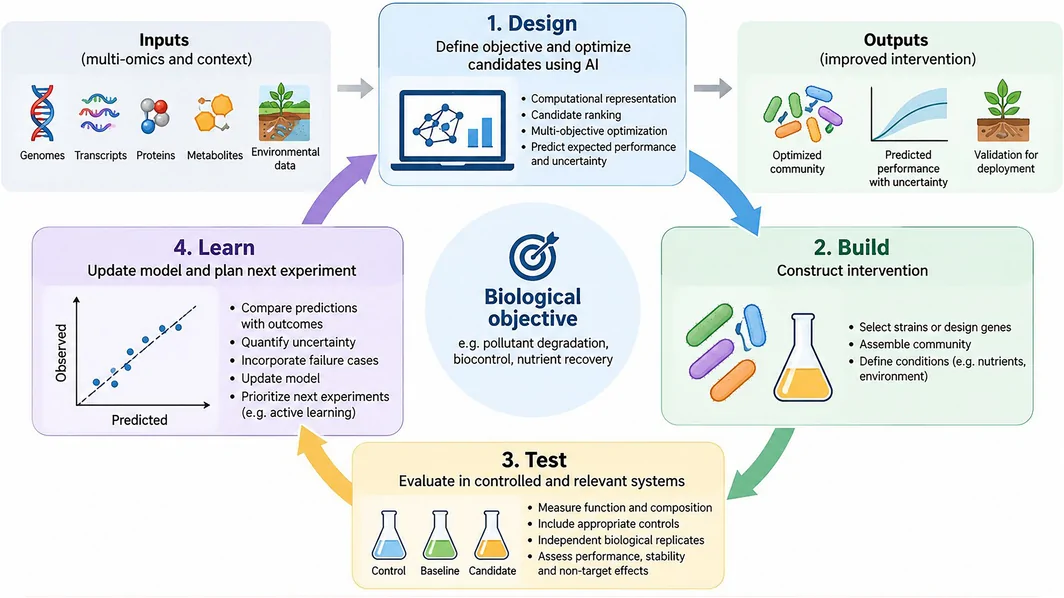
AI‐enabled design–build–test–learn cycle. A predefined biological objective and explicit performance, stability, and safety constraints guide candidate ranking. Interventions are constructed and tested against appropriate controls; observed outcomes and failures inform model updating and subsequent experiments. Mechanistic plausibility, reproducibility, ecological stability, and safety constrain each stage.

## 6 Environmental Microbiology From Controlled Consortia to Open Ecosystems

Environmental systems are difficult to engineer because soil, sediments, wastewater and plant‐associated habitats vary in pH, temperature, moisture, redox conditions, nutrient availability, and indigenous community composition. These factors influence substrate accessibility, microbial interactions, and functional persistence. Consequently, performance in a laboratory assay does not establish efficacy in an environmental setting. Validation must address target function, environmental variability, persistence, and effects on resident communities.

### 6.1 Environmental Validation: The GENIA Example

GENIA illustrates AI‐guided community design for pollutant degradation. De la Vega‐Camarillo et al. (2026) screened 2155 bacterial strains isolated from xenobiotic‐enriched cotton detritusphere and used genome‐informed computational selection to construct a nine‐member consortium. The study reported degradation of lignin, atrazine, and perfluorooctanesulfonic acid (PFOS) under controlled conditions and evaluated the consortium in soil microcosms, reporting more than 70% degradation at 3 weeks.

These findings connect computational selection with measured environmental function, but the evidence remains bounded by the tested conditions. Soil‐microcosm results do not establish field‐scale efficacy, long‐term persistence, or transferability across soil types and climates. Pollutant disappearance also does not necessarily establish complete detoxification; transformation products and residual toxicity may require separate assessment. GENIA therefore demonstrates progress towards environmental microbiome engineering, not a general solution to ecosystem programmability.

### 6.2 A Staged Validation Pathway

Environmental validation should progress from controlled testing to increasingly representative settings. The proposed pathway comprises:

1. Controlled functional testing: Measure the predefined target outcome using suitable controls and biological replication.
2. Environmental sensitivity testing: Assess performance across relevant variation in soil or water chemistry, temperature, moisture, nutrients, and pollutant concentration.
3. Persistence and stability testing: Track community composition, abundance, and function over time and after relevant disturbances.
4. Ecological and risk assessment: Evaluate non‐target effects, changes in resident‐community function, dispersal, and potentially harmful transformation products.
5. Contained mesocosm or field‐relevant evaluation: Advance only when prior evidence supports an acceptable benefit–risk profile, using independent sites where transferability is required.

This is a proposed evidence pathway, not a universal regulatory protocol. The appropriate endpoints and decision thresholds depend on the organism, intervention, and receiving environment.

### 6.3 Biocontrol and Plant‐Associated Communities

Microbial biocontrol may depend on resource competition, antagonistic metabolites, niche occupation or host‐mediated effects. Their relative contributions can vary with plant genotype, soil conditions and indigenous rhizosphere communities. AI may help prioritise candidate consortia by integrating strain traits, environmental measurements and intervention outcomes, but recommendations require external validation across relevant sites, seasons or agricultural conditions. Models should report the environmental domain in which their predictions have been evaluated rather than assume transferability. Research on agricultural SynComs highlights both their potential for soil health and disease suppression and the continuing challenges of assembly and ecological stability (Zhang et al. 2024, 2026).

### 6.4 Persistence, Environmental Release and Biosafety

Environmental deployment raises concerns beyond efficacy, including unintended persistence, dispersal, interactions with resident organisms, and possible genetic transfer. Chemla et al. (2025) discuss the technical and regulatory challenges of environmental release of engineered bacteria. These considerations should inform intervention design rather than be deferred until after candidate selection.

Safeguards may include appropriate containment, persistence and dispersal monitoring, non‐target assessment, and predefined criteria for stopping or modifying an intervention. Risk assessment must reflect the actual organism, engineering strategy, and receiving environment; engineered strains and non‐engineered SynComs should not automatically be assigned identical risk profiles.

The central distinction is between designability and deployability. A candidate may perform under controlled conditions yet remain unsuitable for environmental use because its function is unstable, its performance does not transfer, or its ecological risks are insufficiently characterised.

## 7 Methodological Standards for Trustworthy Ecosystem Prediction

### 7.1 Preventing Data Leakage and Respecting Dependence

All data‐dependent preprocessing, including imputation, normalisation, feature selection, dimensionality reduction, batch correction, and hyperparameter tuning should be fitted within training folds. Held‐out test data must remain independent of model selection to prevent optimistic performance estimates.

Data splitting must also reflect biological dependence and intended use. Longitudinal observations from the same subject or community should remain in the same fold when predicting new subjects or communities. For cross‐study or environmental transfer, grouped or leave‐one‐study‐out validation may be more informative than random splitting. Random splits can be appropriate for narrower prediction tasks, but do not alone demonstrate transferability across sites or studies (Pasolli et al. 2016; Marcos‐Zambrano et al. 2021; Papoutsoglou et al. 2023).

### 7.2 Baselines, Metrics and Uncertainty

Complex AI models should be compared with appropriate statistical, ecological, or mechanistic baselines. Metrics must match the prediction task: classification requires discrimination and, where probabilities guide decisions, calibration; temporal prediction requires errors across forecast horizons; and functional prediction requires target‐specific performance measures. Results should include appropriate uncertainty estimates and biological variability.

Calibrated uncertainty is especially important when predictions guide interventions. Prediction intervals should be evaluated against observed outcomes under the conditions for which the model is intended to be used. Calibration in one environment does not guarantee calibration elsewhere. Models should therefore be assessed for out‐of‐domain predictions, and uncertainty should inform whether to proceed, collect more data, or restrict the operating domain (Papoutsoglou et al. 2023).

### 7.3 Batch Effects, Sequencing Depth and Missing Data

Batch correction can reduce technical variation but may remove genuine biological signals when batch and phenotype are confounded. Studies should document technical metadata, correction procedures, and sensitivity to analytical choices. Sequencing depth and low‐biomass effects can influence rare‐taxon detection; sensitivity analyses should assess whether conclusions remain robust to reasonable filtering and normalisation choices.

Missingness also requires biological interpretation. Random technical failures differ from missing observations associated with community collapse or treatment‐induced changes. Researchers should report missing‐data patterns, justify handling procedures, and test whether conclusions depend on imputation assumptions (Gloor et al. 2017; D'Urso and Broccolo 2024; Papoutsoglou et al. 2023).

Table 2 consolidates these methodological risks and their minimum safeguards, including leakage prevention, dependence‐aware splitting, compositional analysis, baseline comparisons, uncertainty calibration, causal validation, stability testing, transparent reporting, and biosafety assessment. These safeguards should be selected according to the study design and intended application; no single procedure resolves every source of bias.

**TABLE 2:** Methodological failure modes and minimum safeguards for AI‐enabled microbiome prediction.

Failure mode	Consequence for prediction	Minimum safeguard	References
Data leakage	Inflated test performance and unreliable estimates of generalisation	Fit preprocessing, feature selection, and tuning within training folds; preserve an untouched test set	Pasolli et al. (2016); Papoutsoglou et al. (2023)
Dependent observations	Information leakage across time points, replicates, sites or related samples	Split data by subject, community, site, reactor or study according to the intended deployment setting	Marcos‐Zambrano et al. (2021); Papoutsoglou et al. (2023)
Compositionality	Spurious correlations and misleading interpretations of relative‐abundance changes	Use appropriate compositional methods; document zero handling; measure absolute abundance where feasible	Gloor et al. (2017)
Batch effects and domain shift	Models learn technical or site‐specific signatures rather than transferable biological relationships	Report technical metadata; assess confounding; use independent and leave‐one‐study‐out validation where appropriate	Pasolli et al. (2016); D'Urso and Broccolo (2024)
Inadequate baselines	Complex models appear superior without demonstrating meaningful added value	Compare with simple statistical, ecological and mechanistic baselines appropriate to the target	Papoutsoglou et al. (2023); Faure et al. (2023)
Poor uncertainty calibration	Overconfident predictions and unreliable decisions outside the validated domain	Report uncertainty intervals, assess calibration and flag out‐of‐domain predictions	Papoutsoglou et al. (2023)
Causal overclaiming	Associations or feature importance are mistaken for actionable biological drivers	Use controlled perturbations, appropriate comparators, replication and mechanistic follow‐up	Lopatkin and Collins (2020); Papoutsoglou et al. (2023)
Ecological or evolutionary instability	Intended function declines or community composition changes after intervention	Conduct longitudinal stress tests; assess persistence and functional stability; use serial passage where appropriate	Lopatkin and Collins (2020); De la Vega‐Camarillo et al. (2026)
Selective reporting	Successful candidates overrepresent actual model performance	Report evaluated candidates, exclusions, failures and predefined outcomes	Papoutsoglou et al. (2023)
Missing data and informative missingness	Imputation obscures instability or systematically biases predicted trajectories	Describe missingness patterns and plausible causes; perform sensitivity analyses under alternative assumptions	Papoutsoglou et al. (2023)
Safety and persistence	Unintended spread, non‐target effects or ecological disruption	Include containment where appropriate, persistence measurements, non‐target monitoring and risk assessment	De la Vega‐Camarillo et al. (2026)

### 7.4 Prospective Validation and Reproducible Reporting

Models intended to guide intervention should be evaluated prospectively whenever feasible. Predictions, candidate designs, and decision rules should be recorded before outcomes are observed. Experiments should use predefined functional endpoints, suitable controls, and independent biological replicates, with environmental variation relevant to the intended application.

Reproducible reporting should include strain identities and provenance, experimental conditions, inoculation ratios, sampling schedules, functional assays, sequencing methods, exclusions, model versions, preprocessing procedures, and data or code availability. Failed predictions and unsuccessful candidates should be reported alongside positive outcomes to reduce selective reporting and provide information for model refinement. Environmental studies should additionally assess persistence, non‐target effects, and functional changes over time.

Table 3 defines proposed operational criteria for a programmable microbial ecosystem: prospective predictability, perturbational support, controllability, reproducibility, stability, transferability, calibrated uncertainty, ecological and biosafety assessment, and closed‐loop learning. These criteria are a framework proposed by this review, not an established consensus standard. Each criterion should be assessed separately; no cited study should be assumed to satisfy all criteria merely because it demonstrates one component.

**TABLE 3:** Proposed operational criteria for a programmable microbial ecosystem.

Criterion	Minimum evidence required	Interpretation and assessment	References
Prospective predictability	A defined target and a time‐stamped prediction recorded before the outcome is observed	Distinguishes prospective predictive performance from retrospective model fit	Zhang et al. (2025); Papoutsoglou et al. (2023)
Perturbational support	Controlled experiments test the predicted response to a specified intervention	Establishes whether predicted relationships survive experimental manipulation	Lopatkin and Collins (2020); Moyne et al. (2026)
Controllability	An intervention moves the system towards a predefined functional target relative to an appropriate comparator	Demonstrates more than forecasting or classification; requires a measurable intervention‐linked outcome	Moyne et al. (2026); De la Vega‐Camarillo et al. (2026)
Reproducibility	Independent biological replicates or experiments reproduce the principal functional outcome	Reduces dependence on isolated experimental successes	Papoutsoglou et al. (2023); De la Vega‐Camarillo et al. (2026)
Stability	The target function persists over a predefined period and under specified, relevant perturbations	Distinguishes transient responses from sustained performance	Lopatkin and Collins (2020); De la Vega‐Camarillo et al. (2026)
Transferability	The model or intervention is evaluated in an independent community, site, host or environmental condition relevant to the intended use	Defines the conditions under which the prediction can reasonably be generalised	Zhang et al. (2025); De la Vega‐Camarillo et al. (2026)
Calibrated uncertainty	Prediction intervals or probabilities are evaluated against observed outcomes, with performance assessed under the claimed operating conditions	Supports risk‐aware decisions and identifies limitations of model confidence	Papoutsoglou et al. (2023)
Ecological and biosafety assessment	Relevant persistence, non‐target effects and potential environmental risks are measured	Treats ecological safety as part of engineering performance rather than an afterthought	De la Vega‐Camarillo et al. (2026)
Closed‐loop learning	Experimental outcomes update the model and influence subsequent predictions or design decisions	Demonstrates iterative learning rather than a one‐time computational design	Lopatkin and Collins (2020); Moyne et al. (2026)

Finally, Figure 3 integrates these requirements into the five‐layer observe–infer–predict–design–learn framework. Observation establishes the biological and environmental data; inference identifies patterns and candidate mechanisms; prediction estimates outcomes under specified conditions; design selects interventions for a predefined target; and learning uses experimental outcomes to update models and guide subsequent decisions. The feedback loop emphasises that predictive microbiome engineering requires experimental evaluation and model revision rather than computational selection alone.

**FIGURE 3:**
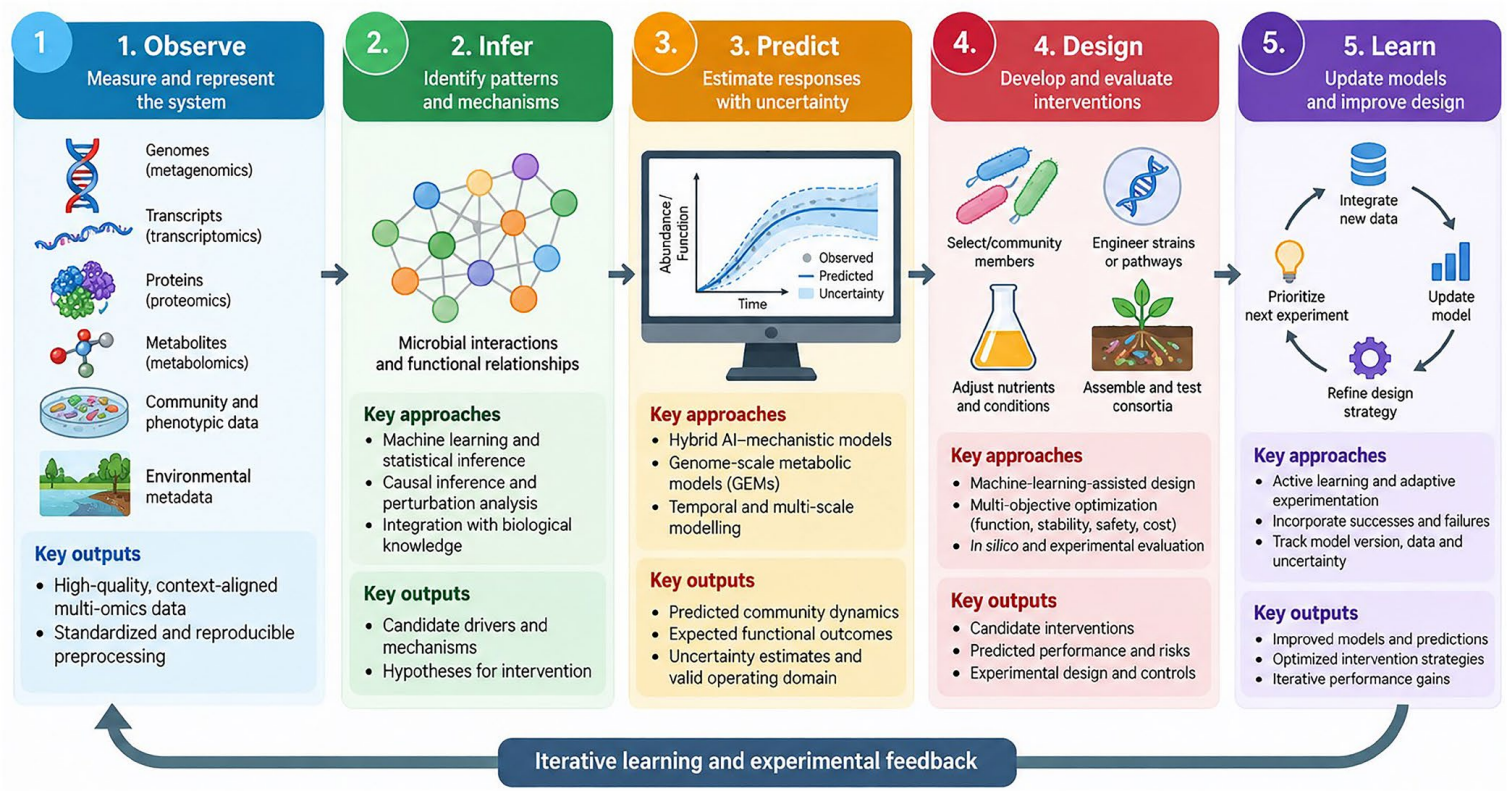
Five‐layer framework for predictive microbiome engineering. The observe–infer–predict–design–learn framework connects biological measurement with iterative experimental improvement. Each layer has distinct evidence requirements, while feedback from experiments informs subsequent predictions and designs. The framework is a proposed organising and evaluation approach, not evidence that all five layers have been validated together in complex natural microbiomes.

## 8 Evidence Synthesis: What Is Demonstrated and What Remains Open

The evidence base for AI‐enabled microbiome engineering is developing unevenly across biological scales. Computational methods are increasingly effective at sequence interpretation, functional annotation, metagenomic classification, and selected metabolic predictions. However, the ability to infer information from molecular data should not be equated with the ability to predict how a microbial community will behave, respond to an intervention, or maintain a target function under changing environmental conditions. These are distinct prediction tasks that require different forms of validation.

At the molecular level, deep‐learning approaches have expanded protein annotation beyond the limits of conventional sequence‐alignment methods. Bileschi et al. (2022), for example, demonstrated that deep‐learning models could assign functional annotations to protein sequences that were difficult to annotate using alignment‐based approaches, increasing coverage of the Pfam protein‐family resource. Likewise, benchmarking of deep‐learning variant callers for bacterial nanopore sequencing has demonstrated improvements in variant discovery under the evaluated conditions (Hall et al. 2024). These advances strengthen the observation and inference layers of predictive microbiome engineering: they help researchers characterise microbial genomes and prioritise candidate functions. Nevertheless, sequence‐derived annotations remain hypotheses about biological function rather than direct evidence that a gene is expressed, that its product is active in a particular environment, or that the corresponding phenotype will emerge within a community. Experimental confirmation and evaluation under relevant environmental conditions therefore remain necessary.

Metagenomic ML provides a second level of evidence. DeepARG, developed by Arango‐Argoty et al. (2018), demonstrated a deep‐learning approach for identifying antibiotic‐resistance genes from metagenomic data, supporting the use of computational tools in resistance surveillance and environmental monitoring. Pasolli et al. (2016) evaluated machine‐learning approaches across large metagenomic datasets and highlighted both the potential of microbiome‐based prediction and the challenges of generalising models across independent studies. Together, these studies establish the usefulness of metagenomic features for defined classification and gene‐prediction tasks, but they do not establish that detected genes will necessarily produce a corresponding phenotype under field conditions. Gene presence, expression, biochemical activity, and ecosystem‐level effects are different evidential endpoints. Predictions of resistance genes, for example, should not be interpreted as direct measurements of resistance phenotype or as proof of a particular environmental transmission outcome.

A further step towards functional prediction is provided by hybrid metabolic modelling. Genome‐scale metabolic models encode biochemical reactions and constraints, while data‐driven methods can help estimate poorly characterised parameters or improve predictions. Faure et al. (2023) demonstrated a neural–mechanistic hybrid approach designed to improve the predictive power of genome‐scale metabolic models. Related machine‐learning methods have also been developed to infer metabolic pathways from tandem mass spectrometry data, including the MS2MP framework (Gonçalves et al. 2023; Bao et al. 2025). These approaches help bridge molecular measurements and metabolic hypotheses. Their outputs, however, remain conditional on model assumptions, training data, reaction‐network completeness, and the environmental conditions represented. A pathway or flux prediction for an organism does not automatically predict the net activity of a community in which resource competition, cross‐feeding, inhibition, spatial structure, and changing physicochemical conditions may alter the outcome.

The most direct evidence for moving from computational inference towards community‐level prediction comes from recent studies that address different parts of the engineering problem. These studies are best interpreted as complementary demonstrations rather than as interchangeable evidence of general microbiome programmability.

MicroProphet: forecasting community trajectories. Zhang et al. (2025) introduced MicroProphet, a time‐aware Transformer framework for forecasting microbial abundance trajectories from incomplete longitudinal observations. The study evaluated the framework using synthetic microbial communities, human gut microbiome data, infant gut development, and postmortem microbial succession. In the synthetic‐community evaluation, the model forecast future trajectories using an initial portion of the observed time series and was benchmarked against alternative statistical and machine‐learning approaches. This provides evidence that data‐driven models can forecast selected aspects of microbial dynamics from sparse temporal observations. It does not, by itself, demonstrate that the model can select an intervention that reliably moves a community towards a specified function. Forecasting observed trajectories and predicting the consequences of a new perturbation are distinct tasks. Moreover, performance across several datasets should not be interpreted as universal transferability across environmental microbiomes because the sampled ecosystems, measurement procedures, and prediction targets define the scope of validation.

MIND: predicting competition and substrate preference. Moyne et al. (2026) developed microbial interaction and niche determination (MIND), which uses translation‐related functional measurements to infer substrate preferences and competitive interactions. The study applied the approach to synthetic communities, soil, human faecal samples and a mouse model, and reported that predictions could guide targeted prebiotic and probiotic interventions. This is important because it links functional measurements to ecological interactions and intervention testing, moving beyond the prediction of community composition alone. Its demonstrated scope should nevertheless be stated precisely: the study focuses on competition and substrate utilisation. It does not establish that every relevant interaction in a complex microbiome can be inferred from the same measurements, or that the predicted effects will remain stable across all host, soil or environmental contexts. Further validation is required for additional ecological mechanisms, longer‐term stability and transfer to settings not represented in the study.

GENIA: genome‐informed consortium design and pollutant degradation. De la Vega‐Camarillo et al. (2026) presented GENIA (Genomically and Environmentally Networked Intelligent Assemblies), a genome‐informed, machine‐learning‐guided framework for designing synthetic microbial communities for pollutant degradation. The study combined high‐throughput cultivation and pollutant‐specific growth screening with genomic and functional information to select a nine‐member consortium. The authors reported degradation of lignin, atrazine, and perfluorooctanesulfonic acid (PFOS) under laboratory conditions and further evaluated the consortium in soil microcosms, where more than 70% degradation was reported at 3 weeks. This is a particularly relevant environmental‐microbiology example because it links computational community selection to measurable bioremediation outcomes beyond an in silico prediction. However, successful performance in laboratory media and soil microcosms is not equivalent to demonstrated efficacy at field‐scale. The latter requires additional assessment of site‐specific chemistry, competing indigenous communities, pollutant bioavailability, seasonal variability, persistence, non‐target effects, and the reproducibility of function across independent sites.

These three studies therefore address different targets and provide different kinds of evidence. MicroProphet primarily addresses temporal forecasting; MIND addresses functional inference of competition and resource use, with intervention testing; and GENIA addresses computational selection of a consortium followed by experimental evaluation of pollutant degradation. Their combined significance is not that a universal programmable microbiome has already been achieved. Rather, they show that specific capabilities within the observe–infer–predict–design–learn framework can be tested and demonstrated under defined conditions.

A central unresolved issue is whether these capabilities can be integrated into a reproducible, prospective, and genuinely iterative engineering process. A convincing demonstration would require investigators to define a target ecosystem function before the experiment; generate and record a prediction before observing the outcome; choose an intervention based on that prediction; test the intervention against suitable controls; quantify the uncertainty and variability of the response; and reproduce the effect in independent communities or environmental contexts. The programme should also establish whether the desired function persists over a specified period and under relevant disturbances, including changes in nutrient supply, temperature, pH, moisture, pollutant availability, or community composition. Where appropriate, longitudinal experiments should assess whether evolutionary change alters the function or undermines the assumptions of the original model.

The distinction between predictive performance and ecological deployability is particularly important for environmental applications. A model may accurately predict community composition yet poorly predict the function that matters for a remediation or bioprocess objective. Conversely, a consortium may perform well in a controlled experiment without maintaining the same function in a field environment. Relative‐abundance profiles alone may also obscure changes in absolute microbial abundance, while technical batch effects, compositional data, uneven sequencing depth, and non‐independent observations can inflate apparent model performance if they are not handled appropriately. Robust evaluation should therefore use baselines suited to the task, separate training and testing at the appropriate biological or environmental unit, report uncertainty and failure cases, and test transferability explicitly rather than infer it from internal validation.

Overall, the evidence supports a calibrated conclusion. AI is already useful for molecular annotation, metagenomic classification, selected metabolic predictions, and increasingly sophisticated analyses of microbial communities. Recent studies also demonstrate meaningful progress towards forecasting microbial trajectories, predicting specific ecological interactions, and designing pollutant‐degrading consortia. Yet these achievements do not independently establish the full programmability of complex microbial ecosystems. That stronger claim requires evidence of prospective prediction, intervention‐linked control, reproducibility, stability, transferability, calibrated uncertainty, and ecological safety. The most productive next step is therefore not simply to develop more complex algorithms, but to design experiments that evaluate the complete prediction–intervention–measurement–model‐update loop.

## 9 Research Priorities

Progress in AI‐enabled predictive microbiome engineering should follow a staged validation programme that advances from reliable measurement to environmentally relevant application. The five stages below establish progressively stronger evidence requirements.

Stage 1: Standardised measurement and transparent baselines. Establish reproducible sampling, sequencing, multi‐omics workflows, and metadata reporting. Document sequencing depth, batch effects, environmental conditions, and data‐processing procedures. Benchmark AI models against appropriate statistical and mechanistic baselines before attributing improvements to algorithmic complexity.

Stage 2: Prospective prediction in defined communities. Evaluate predictions in controlled synthetic microbial communities with specified membership, initial conditions, and measurable functional outcomes. Predictions should be recorded before experimental results are known, and evaluation should use independent biological replicates and appropriate validation splits.

Stage 3: Perturbation‐based mechanism testing. Use controlled experiments to determine whether predicted microbial interactions or candidate drivers produce the expected responses to changes in substrates, environmental conditions, community composition, or targeted interventions. Distinguish predictive associations from causal mechanisms and report negative as well as positive results.

Stage 4: Reproducibility, stability and transferability. Assess performance across independent communities, experimental batches, time points and relevant environmental conditions. Quantify prediction uncertainty, evaluate calibration and identify conditions under which models fail. Longitudinal measurements should establish whether community composition and target function persist after perturbation.

Stage 5: Field‐relevant validation and safety. Progress from laboratory experiments to mesocosms and other field‐relevant systems before considering deployment. Evaluate target‐function performance alongside non‐target effects, ecological persistence, community displacement, and other application‐specific risks. Environmental performance should be demonstrated under realistic variability rather than inferred from success in a defined laboratory medium.

Progression between stages should depend on evidence generated at the preceding stage, not simply on improvements in model complexity or internal predictive accuracy. This staged approach provides a practical route for translating computational predictions into experimentally supported and environmentally responsible microbial engineering.

## 10 Conclusion

AI is expanding the capacity to interpret microbial sequences, integrate multi‐omics data, predict metabolic properties and guide the design of microbial communities. Nevertheless, the ability to represent a microbial system computationally does not, by itself, establish that the system can be predicted reliably or controlled through intervention. The distinction is especially important in environmental microbiology, where ecological interactions, spatial structure, resource availability, and changing environmental conditions can alter community composition and function. The proposed observe–infer–predict–design–learn framework connects data acquisition, biological interpretation, prospective modelling, intervention design, and experimental feedback to explicit standards of evidence. Recent studies demonstrate meaningful progress in microbial trajectory forecasting, prediction of competition and substrate preferences, and AI‐guided environmental consortium design. These advances remain specific to their experimental systems and validation conditions and should not be interpreted as universal evidence of programmable natural ecosystems. Programmability should therefore be treated as an empirical property requiring prospective prediction, perturbational support, reproducibility, stability, transferability, calibrated uncertainty, and safety. The central challenge is not merely to build more sophisticated algorithms, but to establish when, where, and why their predictions remain reliable under biological and environmental change. Integrating AI with mechanistic models, well‐designed perturbation experiments, and iterative validation offers a rigorous path towards microbial engineering that is both functionally effective and ecologically responsible.

## Author Contributions

**Mohammad Nazrul Islam Bhuiyan:** conceptualization, writing – original draft, writing – review and editing. **Sheikh Julfikar Hossain:** writing – review and editing.

## Funding

The authors have nothing to report.

## Conflicts of Interest

The authors declare no conflicts of interest.

## Data Availability

Data sharing not applicable to this article as no datasets were generated or analysed during the current study.
